# Influenza T-cell Epitope-Loaded Virosomes Adjuvanted with CpG as a Potential Influenza Vaccine

**DOI:** 10.1007/s11095-014-1556-3

**Published:** 2014-10-25

**Authors:** Peter C. Soema, Sietske K. Rosendahl Huber, Geert-Jan Willems, Wim Jiskoot, Gideon F. A. Kersten, Jean-Pierre Amorij

**Affiliations:** 1 Intravacc (Institute for Translational Vaccinology), Antonie van Leeuwenhoeklaan 9, 3721 MA Bilthoven, The Netherlands; 2 Division of Drug Delivery Technology, Leiden Academic Centre for Drug Research (LACDR), Leiden University, Einsteinweg 55, 2333 CC Leiden, The Netherlands; 3 Centre for Infectious Disease Control Netherlands, National Institute for Public Health and the Environment (RIVM), Antonie van Leeuwenhoeklaan 9, 3721 MA Bilthoven, The Netherlands

**Keywords:** cross-protective, cytotoxic T-cells, influenza vaccine, peptide vaccine, virosomes

## Abstract

**Purpose:**

Influenza CD8^+^ T-cell epitopes are conserved amongst influenza strains and can be recognized by influenza-specific cytotoxic T-cells (CTLs), which can rapidly clear infected cells. An influenza peptide vaccine that elicits these CTLs would therefore be an alternative to current influenza vaccines, which are not cross-reactive. However, peptide antigens are poorly immunogenic due to lack of delivery to antigen presenting cells, and therefore need additional formulation with a suitable delivery system. In this study, the potential of virosomes as a delivery system for an influenza T-cell peptide was investigated.

**Methods:**

The conserved human HLA-A2.1 influenza T-cell epitope M1_58–66_ was formulated with virosomes. The immunogenicity and protective effect of the peptide-loaded virosomes was assessed in HLA-A2 transgenic mice. Delivery properties of the virosomes were studied in mice and in *in vitro* dendritic cell cultures.

**Results:**

Immunization of HLA-A2.1 transgenic C57BL/6 mice with peptide-loaded virosomes in the presence of the adjuvant CpG-ODN 1826 increased the number of peptide-specific CTLs. Vaccination with adjuvanted peptide-loaded virosomes reduced weight loss in mice after heterologous influenza infection. Association with fusion-active virosomes was found to be crucial for antigen uptake by dendritic cells, and subsequent induction of CTLs in mice.

**Conclusions:**

These results show that influenza virosomes loaded with conserved influenza epitopes could be the basis of a novel cross-protective influenza vaccine.

**Electronic supplementary material:**

The online version of this article (doi:10.1007/s11095-014-1556-3) contains supplementary material, which is available to authorized users.

## INTRODUCTION

The need for cross-protective influenza A vaccines has increased in recent years after several global outbreaks of highly pathogenic influenza A strains, such as avian H5N1 (1), swine H1N1 and avian H7N9 (2,3). The current, mainly antibody-inducing influenza A vaccines are generally ineffective against influenza A strains which underwent antigenic shifts and drifts, which leads to vaccine mismatch. Influenza-specific antibodies induced by mismatched vaccines fail to recognize the surface proteins hemagglutinin (HA) and neuraminidase (NA). As a result, the composition of the current influenza vaccines has to be adjusted frequently to cope with these antigenic changes. CD8^+^ cytotoxic T-cells (CTLs) that are specific for conserved epitopes of internal influenza A proteins, such as matrix protein and nucleoprotein, may provide cross-protection and are thus unaffected by antigenic changes (4). Influenza-specific CTLs can efficiently clear virus-infected cells, thereby stopping viral replication and spread. Influenza-specific CD8^+^ T-cells induced by influenza infection were recently correlated with less severe illness in adults infected with pandemic H1N1 virus (5). Inducing influenza-specific CTLs by vaccination could therefore be a promising approach to achieve cross-protection against heterologous influenza A strains (6).

The influenza M1_58–66_ peptide is a highly conserved human major histocompatibility complex (MHC) class I restricted epitope (7,8), which can induce influenza-specific CTLs. However, before CD8^+^ T-cells can be induced, several critical processes have to take place (9). Delivery of the peptide antigens to antigen-presenting cells (APCs), in particular dendritic cells (DCs), is crucial for antigen presentation on MHC class I molecules. Therefore, formulation of the peptide antigens with a suitable delivery system, such as virosomes, is required. Influenza virosomes were previously shown to be capable of efficient delivery of peptide antigens and subsequent CTL induction (10). However, virosomal formulations can only deliver low doses of peptide antigens and lack pathogen-associated molecular patterns (PAMPs). The inclusion of immunopotentiators such as Toll-like receptor (TLR) agonists could improve the immunogenicity of the vaccine formulation, without the need to increase the peptide loading efficiency in virosomes, which requires complicated methods (11). While adjuvants have been used to increase neutralizing antibody levels induced by virosomal vaccines (12), their effect on influenza peptide-specific CD8^+^ T-cell responses in combination with virosomes has yet to be determined.

In this study, we investigated the immunogenicity of virosomes loaded with the human HLA-A2.1-restricted peptide M1_58–66_, derived from influenza matrix protein 1. Influenza-specific CD8^+^ T-cell responses and supporting antibody titers were assessed in HLA-A2.1 transgenic mice after immunization. The addition of TLR9 agonist CpG was found to be an effective adjuvant for MHC class I restricted peptides in conjunction with virosomes. Furthermore, immunization with peptide-loaded virosomes adjuvanted with CpG increased the rate of recovery in mice infected with a heterologous influenza strain. Finally, delivery properties of virosomes were extensively characterized in *in vitro* human DC models and *in vivo* in transgenic mice. Both peptide association with the virosomes and virosomal cell binding and membrane fusion capabilities were found to be crucial for peptide uptake by DCs and induction of peptide-specific CTLs in mice.

## MATERIALS AND METHODS

### Preparation of Virosomes

Virosomes were prepared from β-propiolactone inactivated egg-derived influenza A/PR8/34 H1N1 virus as described previously (13). In brief, whole inactivated influenza virus (WIV) was disrupted by the addition of 100 mM 1,2-dihexanoyl-*sn*-phosphatidylcholine (DHPC, Avanti Polar Lipids) in HNE (5 mM HEPES, 150 mM NaCl, 1 mM EDTA, pH 7.3) buffer. Nucleocapsid was removed from the membrane lipids and surface proteins by ultracentrifugation. Virosomes were reconstituted by removal of the detergent by dialysis against HNE buffer. Subsequently, virosomes were purified by centrifugation on a discontinuous sucrose gradient (10%/60% *w*/*v* in HNE), and sucrose was removed by dialysis against HNE buffer. Peptide-loaded virosomes were obtained by adding 125 μg/mL M1_58–66_ peptide (GILGFVFTL, kindly provided by the Netherlands Cancer Institute) to the virosomes (1:36 peptide:protein *w*/*w* ratio) prior to detergent removal to enable peptide encapsulation. As negative control, fusion-inactivated virosomes were prepared. Virosomes were fusion-inactivated after peptide-loading by lowering the buffer pH to 4.5 with a pretitrated volume of 1 M HCl, and subsequently incubated at 37°C for 15 min. Afterwards, pH was restored to pH 7.3 with a pretitrated volume of 1 M NaOH.

### Characterization of Virosome Formulations

Protein composition of the peptide-loaded virosomes was determined by SDS-PAGE, by using a 12% precast polyacrylamide gel (Thermo Scientific) and Coomassie Brilliant Blue (Thermo Scientific) staining.

Mean particle size distribution and zeta potential were determined by dynamic light scattering (DLS) with a Malvern Nano ZS (Malvern Instruments). Samples were diluted six fold in MilliQ for zeta potential analysis.

### Hemolysis Assay

Virosome fusion activity was determined by using a hemolysis assay as described previously (14). Vaccine preparations were mixed with human blood erythrocytes and 0.1M 2-(N-morpholino)ethanesulfonic acid (MES) buffer with different pH ranging from 4.5 to 5.5, and incubated at 37°C. After allowing fusion for 30 min, the released hemoglobin was quantified in the supernatant after centrifugation by reading absorbance at 540 nm using a Synergy Mx reader (Biotek). Hemoglobin release of erythrocytes mixed with water was set as maximal hemolysis (100%).

### Association of Peptide to Virosomes

The interaction between peptides and virosomes was studied using size-exclusion chromatography (SEC). M1_58–66_ peptide was labeled with fluorescein for detection purposes. Peptide-loaded virosomes or peptide mixed with empty virosomes were applied on a pre-washed PD-10 column (GE Healthcare). Samples were eluted with HNE buffer, and fractions of 1 mL were collected in tubes and subsequently analyzed for peptide and protein content by using fluorescence (exCitation at 480 nm and emission at 530 nm) and Lowry protein assay respectively.

### Immunizations

Animal experiments were performed according to the guidelines provided by the Dutch Animal Protection Act, and were approved by the Committee of Animal Experimentation (DEC) of the National Institute of Public Health and the Environment (RIVM) under registration numbers PO201200156 and PO201300046. 8–12 week old female HLA-A2.1 transgenic C56BL/6 mice (bred and maintained at Intravacc) were used in all studies. Mice received subcutaneous injections (100 μl/dose) in the left hind flank at day 0 and 21 under isoflurane anesthesia. Immunizations consisted of either PBS, peptide mixed with CpG, peptide in 50% Incomplete Freund’s Adjuvant (IFA, Sigma-Aldrich) with CpG, peptide-loaded virosomes (mixed with and without CpG), inactivated peptide-loaded virosomes mixed with CpG, and free peptide mixed with empty virosomes and CpG. Mice received 1 μg of M1_58–66_ peptide per dose and 180 μg of virosomal protein. When mentioned, 50 μg of CpG-ODN 1826 (Invivogen) per dose was used as an adjuvant. As a positive control, one group of mice was infected with 1 × 10^3^ PFU of influenza A/HKx31 H3N2 virus in 50 μl PBS by intranasal administration under isoflurane anesthesia. On day 35 animals were sacrificed by bleeding and cervical dislocation under isoflurane anesthesia.

### Challenge Study

For the challenge study, six mice were immunized as described previously. Additionally, one group of mice was immunized twice with 180 μg (total protein) of influenza A/PR8 WIV vaccine on the same immunization schedule as previous groups. On day 35, mice were infected with a sublethal dose of 1 × 10^5^ PFU of influenza A/HKx31 virus in 50 μl PBS by intranasal administration. Subsequently, mice were weighed daily until day 14 after challenge, after which the mice were sacrificed.

### Intracellular Staining

Peptide-specific cytotoxic T-cells were quantified by flow cytometry analysis. Single cell suspensions of excised spleens were obtained using 70 μm cell strainers (BD Falcon). Subsequently, 2*10^6^ splenocytes were plated per well in 48-wells culture plates (Greiner) and restimulated with 50 ng M1_58–66_ peptide for 18 h at 37°C 5% CO_2_. Next, Golgi-plug (1:1000, BD) was added to the cells to inhibit cellular protein and cytokine transport, and cells were incubated for another 4 h. Subsequently, cells were transferred to a 96-wells plate, washed twice with FACS buffer (PBS, 0.5% BSA), and stained with anti-mouse CD4-PE (BD Biosciences), anti-mouse CD8-FITC (BD Biosciences) and Live/dead-Aqua (Invitrogen). Next, cells were washed twice with FACS buffer, and fixed with fixation-permeabilization buffer (BD Biosciences). Subsequently cells were washed with permeabilization wash buffer (BD Biosciences), and intracellular staining was performed with IFN-γ-APC (Biolegend). Finally, cells were washed with FACS buffer and 1.5 to 2 million cells were measured on a FACS Canto II flow cytometer (BD Biosciences). Data was analyzed using FlowJo software version 9 (Tree Star) for Mac OSX.

### Enzyme Linked Immunosorbent Spot Assay (ELISpot)

Cytokines produced by spleen cells were determined by ELISpot. 96-wells Multiscreen PVDF filter plates (Millipore) were activated by adding 25 μL 70% ethanol for 2 min, and subsequently washed three times with PBS. Plates were coated overnight with anti-mouse IFN-γ antibodies (U-Cytech) at 4°C. Next, filter plates were washed three times and blocked with 5% Hyclone fetal calf serum (FCS, Thermo Scientific) for 1 h at 37°C. Subsequently, 4*10^5^ isolated spleen cells in IMDM medium, 5% FCS were added to each well with or without 50 ng M1_58–66_ peptide, and incubated overnight at 37°C. After overnight stimulation, filter plates were washed five times and IFN-γ was detected using biotinylated anti-mouse antibodies (U-Cytech) and 100 μL BCIP/NBT reagent (Thermo Scientific) per well. Spots were allowed to develop for 15 min after which the plates were thoroughly washed with tap water. Spots were counted using an A.EL.VIS ELISpot reader (Aelvis). The number of IFN-γ producing cells in antigen stimulated spleen cells was obtained after background correction (subtracting number of spots produced by splenocytes incubated with medium).

### Hemagglutination Inhibition Assay

Hemagglutination-inhibition (HI) titers in mouse sera were determined by a HI assay. Sera were treated overnight with diluted receptor-destroying enzyme from *Vibrio cholerae* (1:5, Sigma-Aldrich) at 37°C to remove non-specific inhibitors, and were subsequently inactivated at 56°C for 30 min. Finally, PBS was added to the sera to obtain a 1:10 dilution. Diluted sera were serially diluted two-fold with PBS. Four hemagglutinating units of inactivated influenza A/PR/8/34 or influenza A/HKx31 were subsequently added to each well and incubated for 20 min at room temperature after mixing. Next, an equal amount of a 0.5% (*v*/*v*) turkey erythrocyte suspension was added to the wells and incubated for 45 min at room temperature. HI titers are reported as the reciprocal of the highest serum dilution capable to completely prevent hemagglutination.

### Enzyme Linked Immunosorbent Assay (ELISA)

Influenza A/PR8 and A/HKx31-specific antibody titers were determined by ELISA as described previously (15). In short, Microlon 96-wells flatbottom plates (Greiner) were coated overnight with either 600 ng (HA) of A/PR8/34 subunit or A/HKx31 virus per well at 4°C. Serial two-fold dilutions of individual mouse sera in PBS, 0.5% BSA, 0.1% Tween80 were applied on the plate and incubated for 1 h at 37°C. Subsequently, plates were incubated for 1 h at 37°C with horseradish peroxidase-conjugated goat antibodies against mouse IgG, IgG1 or IgG2c (1:5000, Southern Biotech). Detection of antibodies was performed with TMB substrate buffer (0.4 mM TMB in 0.11 M sodium acetate, 0.006% H_2_O_2,_ pH 5.5). The reaction was stopped by adding 2 M sulfuric acid, after which the optical density (OD) was measured at a wavelength of 450 nm by using a Synergy Mx platereader (BioTek). Titers are reported as the reciprocal of the serum dilution corresponding to OD_450_ = 0.2 after background correction.

### Dendritic Cell Uptake Studies

Peptide antigen uptake by DCs was determined as follows. Fresh blood was collected from healthy donors and collected in heparin-coated tubes. Peripheral blood mononuclear cells (PBMCs) were isolated from whole blood using a Lymphoprep (Axis-Shield) gradient. Subsequently, CD14^+^ monocytes were isolated from the PBMC fraction by labeling with human CD14 MicroBeads (Miltenyi Biotec) and subsequent separation with a magnetic LS MACS column (Miltenyi Biotec). Finally, monocytes were plated at a concentration of 0.4*10^6^ cells/mL in 48-wells plates in IMDM medium (Invitrogen) containing 1% FCS, 500 U/mL GM-CSF (Peprotech) and 800 U/mL IL-4 (Sanquin). After 6 days, vaccine formulations were incubated for 4, 24 and 48 h with the immature DCs at a concentration of 50 ng/mL M1_58–66_-FITC peptide in IMDM with 500 U/mL GM-CSF. Subsequently, cells were transferred to a 96-wells plate, washed twice with FACS buffer, and stained with Live/dead-Aqua. Cells were washed twice with FACS buffer and analyzed on a FACS Canto II flow cytometer. Data was analyzed by using FlowJo 9 software for Mac OSX. Peptide uptake is reported as mean fluorescent intensity (MFI) of the FITC signal.

### Statistics

Data were analyzed by using one-way ANOVA with Tukey-Kramer’s method for multiple comparisons. Probability (p) values of *p* ≤ 0.05 were considered statistically significant. Statistics were performed by using Graphpad Prism Software version 6.03 for Windows.

## RESULTS

### Characteristics of Peptide-Loaded Virosomes

**Fig. 1:**
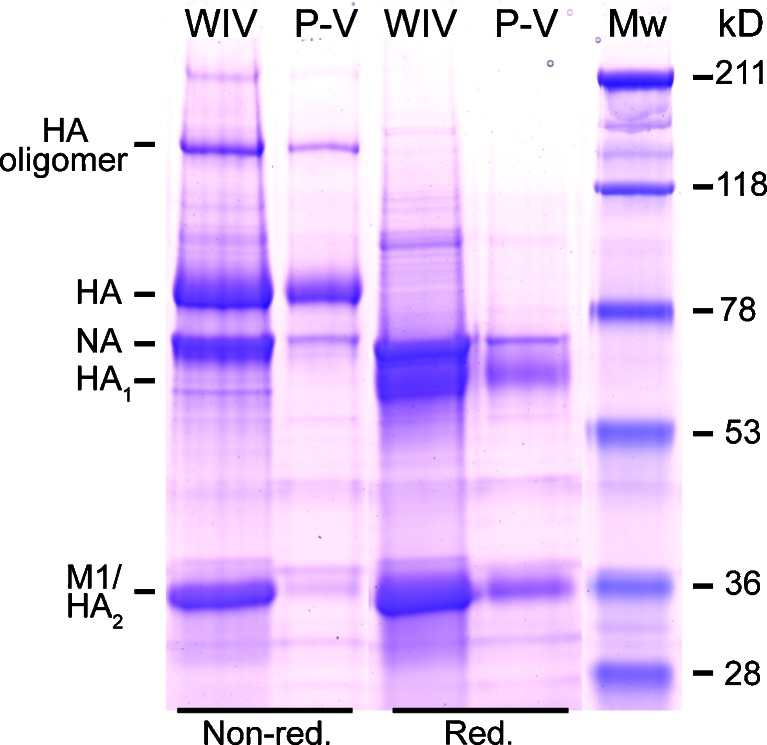
Protein profile of peptide-loaded virosomes. SDS-PAGE analysis of peptide-loaded virosomes (P-V) and source material whole inactivated influenza virus (WIV) under non-reduced and reduced conditions on a 12% precast gel stained with Coomassie Brilliant Blue. Protein identities were confirmed by mass spectrometry.

To confirm that virosome production was successful, the protein composition of peptide-loaded virosomes was analyzed by SDS-PAGE. We observed that peptide-loaded virosomes (P-V) retained HA and NA proteins, whereas internal proteins, such as matrix protein 1 (M1), were removed from the virosome particles (Fig. 1). Additionally, SDS-PAGE analysis of P-V under reducing conditions revealed that subunits HA_1_ and HA_2_ from HA mono- and dimers were formed, similar to WIV. Dynamic light scattering showed a particle size of 140 ± 2 nm (mean ± SD, *n* = 3) for P-V, which was comparable to the size of source material WIV (143 ± 1 nm). The polydispersity index (PDI) of P-V was 0.121, indicating that the particle distribution was very homogeneous and comparable to the PDI of WIV (0.036). The zeta potential of virosomes was −21.2 ± 1.7 mV (mean ± SD, *n* = 3), which was similar to that of WIV (−21.5 ± 0.3 mV). Therefore, the peptide-loaded virosomes closely resembled WIV particles in terms of particle size and surface charge, but were enriched in HA and NA.

**Fig. 2:**
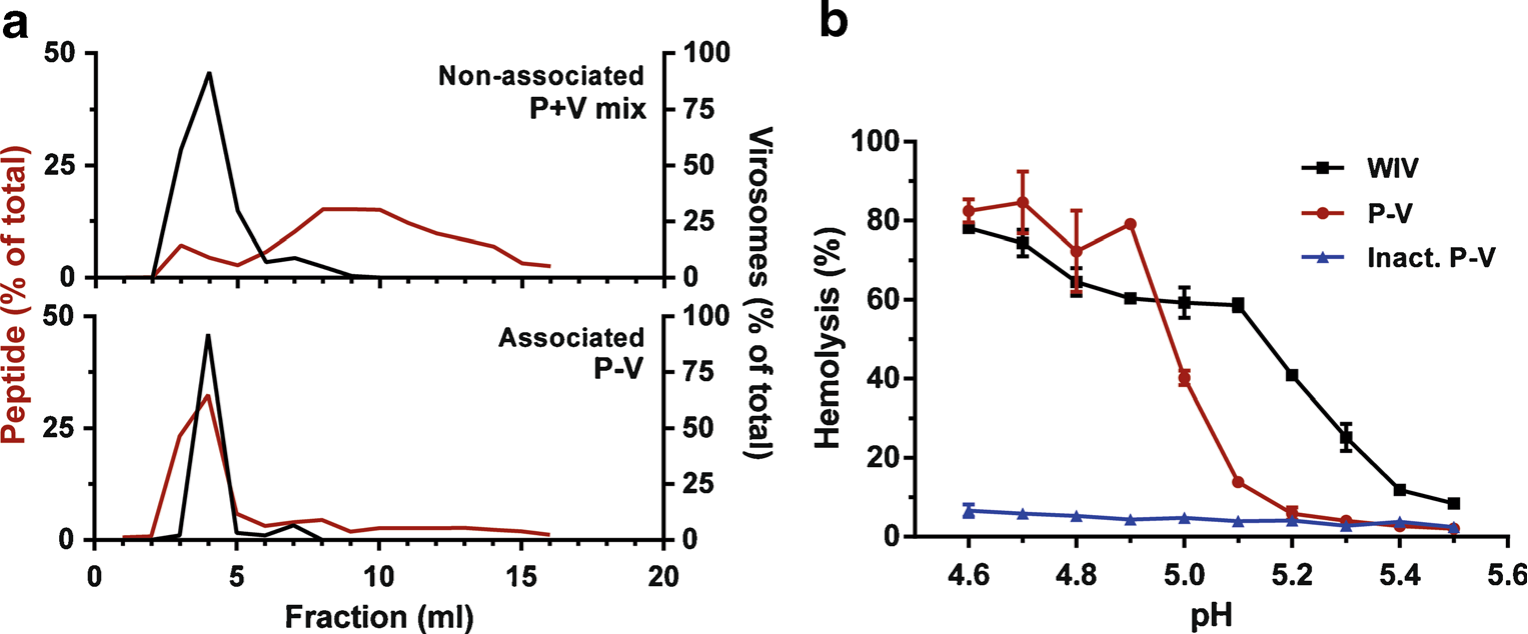
Characteristics of peptide-loaded virosomes. Peptide association of peptide mixed with virosomes (P+V mix) and peptide-loaded virosomes (P-V) analyzed by size exclusion chromatography (**a**). *Black lines* show the virosome elution pattern (based on protein determination), whereas the *red line* shows the elution of peptide (based on fluorescence of M1_58–66_-FITC). The fusogenic activity of WIV (*black*), P-V (*red*) and fusion-inactivated P-V (*blue*) was determined between pH 4.6 and 5.5 by a hemolysis assay (**b**). Data represents mean ± SD (*n* = 3).

Size-exclusion chromatography confirmed that simple mixing of peptide and virosomes did not result in substantial association between the two components (Fig. 2a). As expected, both peptide and virosomes co-eluted when peptide-loaded virosomes were applied onto the SEC column, indicating association. The association efficiency of the peptide with the virosomes was typically 4–6% of the total amount of added peptide.

In order to confirm whether P-V still possessed fusion activity, a hemolysis assay was performed. Both peptide-loaded virosomes and WIV showed low-pH induced fusion activity in the pH range that is representative for the endosome (Fig. 2b). Additionally, P-V were shown to be successfully fusion-inactivated by short (15 min) exposure to pH 4.5. P-V fused at slightly lower pH compared to WIV, which might be caused by small differences in HA confirmation and stability in peptide-loaded virosomes.

### Immunogenicity of Peptide-Loaded Virosomes in HLA-A2 Transgenic Mice

**Fig. 3:**
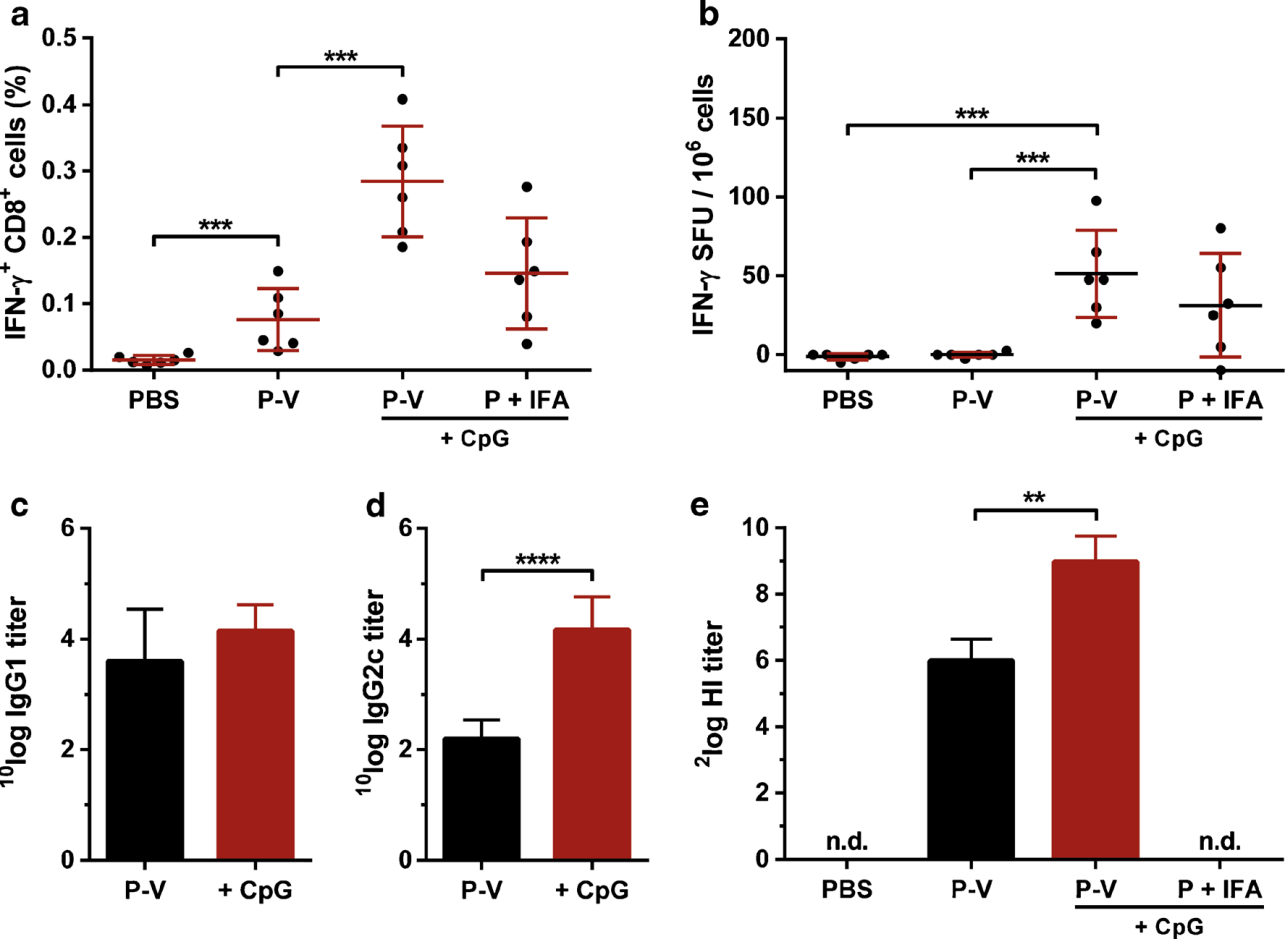
Immunogenicity of (non-)adjuvanted peptide-loaded virosomes. HLA-A2.1 transgenic C57BL/6 mice were immunized twice with 1 μg of M1_58–66_ peptide formulated in either virosomes (P-V), virosomes adjuvanted with CpG (P-V + CpG) or Incomplete Freund’s Adjuvant (IFA) with CpG (P + IFA + CpG). Mice were immunized with PBS as negative control. Two weeks after the final vaccination, peptide-specific CD8+ T-cell responses in *ex vivo* stimulated splenocytes were determined using flow cytometry (**a**) and ELISPOT (**b**). Antibody isotypes IgG1 (**c**) and IgG2c (**d**) titers were determined in mice sera. HI titers were also determined (**e**). Data represents mean ± SD (*n* = 6); *p < 0.05, **p < 0.01, ***p < 0.001, ****p < 0.0001; n.d., not detectable.

To assess whether the produced peptide-loaded virosomes were able to induce peptide-specific T-cell responses, HLA-A2.1 transgenic C57BL/6 mice were primed and boosted three weeks after priming with either peptide-loaded virosomes or P-V adjuvanted with CpG. PBS and peptide mixed with IFA and CpG were administered as negative and positive control, respectively. Peptide-specific T-cell responses in restimulated splenocytes were determined 2 weeks after booster vaccination. Flow cytometry analysis revealed that splenocytes from mice immunized with peptide-loaded virosomes contained peptide–specific IFN-γ^+^ CD8^+^ T-cells after *ex vivo* stimulation (Fig. 3a), as opposed to PBS injected mice. P-V were able to induce peptide-specific IFN-γ^+^ CD8^+^ T-cell levels comparable to the levels induced by P mixed with IFA and CpG. The addition of CpG to P-V significantly (*p* < 0.001) increased the number of peptide-specific CD8^+^ T-cells, which confirmed the immunopotentiating effect of CpG. The increase of IFN-γ^+^ CD8^+^ T-cells after adding CpG to the P-V formulation was in line with the increased frequency of IFN-γ producing cells observed utilizing an ELISPOT assay (Fig. 3b). In order to investigate the contribution of viral material to the T-cell response, mice were immunized with WIV mixed with peptide and CpG. Flow cytometry (Supplementary Figure S1A) and ELISPOT (Supplementary Figure S1B) assays confirmed that WIV induced minimal peptide-specific T-cells.

While virosomes mainly act as an efficient vehicle to deliver the peptide antigen to the APCs, the presence of CD4^+^ T-cell epitopes in the sequence of virosomal surface proteins enable virosomes to provide helper T-cell (T_H_) responses. T_H_ responses are able to support the induction of CD8^+^ T-cells (16), and CD4^+^ T-cell epitopes are thus an important part of the vaccine formulation. To gain further insight into the possible T-cell help that virosomes and the adjuvant provide, the T_H1_/T_H2_ balance was assessed by determination of IgG1 and IgG2c isotype titers induced by P-V with or without CpG. The results show that IgG1 titers remained unchanged (Fig. 3c) after addition of CpG, but IgG2c titers were significantly (*p* < 0.0001) increased after vaccination with CpG adjuvanted P-V when compared to P-V alone (Fig. 3d). This indicates that addition of CpG to P-V skewed influenza specific responses towards a T_H1_ response, which supports the CD8^+^ T-cell response against the influenza peptide.

In addition to the T-cell epitopes, the P-V formulations contain virosomal B-cell epitopes (mainly on surface antigens HA and NA) that induce influenza-specific antibodies. While these antibodies are usually not cross-reactive, they do provide protection against homologous influenza strains. Thus, in case the circulating virus matches the source influenza strain of the virosomes, additional humoral responses can aid in protection. CpG adjuvanted P-V induced significantly (*p* < 0.01) higher HI titers compared to non-adjuvanted P-V (Fig. 3e). Total IgG titers showed a similar significant (*p* < 0.05) increase after addition of CpG to the P-V (Supplementary Figure S1C). This underlines that CpG is not only an effective adjuvant for T-cell induction, but also improves B-cell responses, as observed previously (12). As expected, there were no detectable HI and total IgG titers in sera of control mice receiving PBS or peptide mixed with IFA and CpG, due to the lack of influenza surface antigens in these formulations.

### Efficacy of Peptide-Loaded Virosomes Against Heterologous Influenza Infection in Mice

**Fig. 4:**
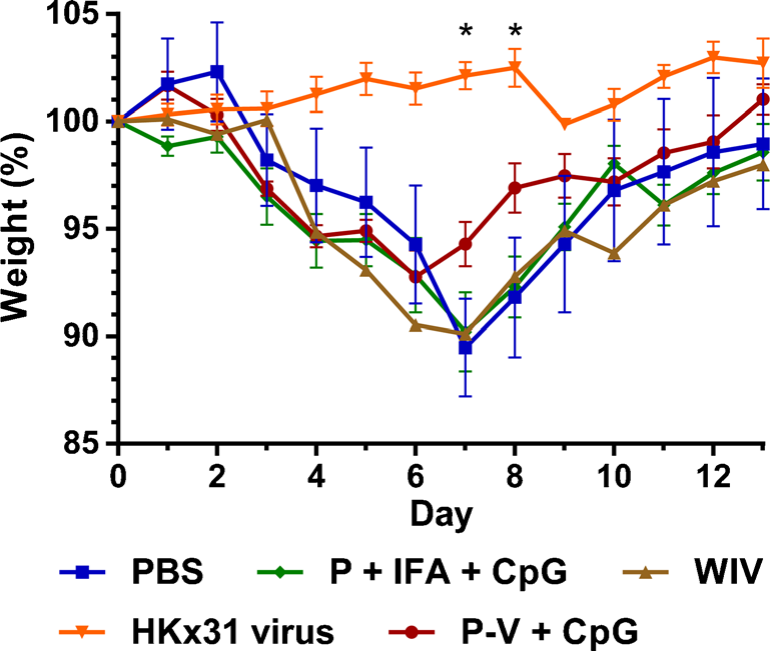
Efficacy of vaccine after sublethal heterologous influenza infection. HLA-A2.1 transgenic C57BL/6 mice were immunized twice with either peptide-loaded virosomes with CpG (*red*), peptide mixed with incomplete Freund’s adjuvant and CpG (*green*), WIV (*brown*) or PBS (*blue*). As a positive control, mice were challenged with a sublethal dose of HKx31 virus. Subsequently, mice were infected with heterologous HKx31 (H3N2) influenza virus and their weight was monitored for 14 days. Data represents mean ± SEM (*n* = 6); *p < 0.0001 for PBS and P + IFA + CpG groups *versus* HKx31.

In addition to the assessment of immunological responses, the efficacy of the P-V vaccine against heterologous influenza infection was examined. HLA-A2.1 transgenic C57BL/6 mice were immunized with the vaccine and subsequently infected with a sublethal dose of influenza HKx31 (H3N2) virus. The weight of the infected animals was monitored for 14 days (Fig. 4). Mice that were previously infected with a small dose of live influenza HKx31 did not show any weight loss after challenge. Mice that received either PBS, WIV or peptide mixed with IFA and CpG showed a decline in weight until day 7, after which the animals recovered slowly. In contrast, mice immunized with CpG adjuvanted P-V started to recover already after day 6, and their total weight loss was less severe than that of mice immunized with PBS or peptide mixed with IFA and CpG. Moreover, at day 7 and 8, the weight of mice immunized with P-V adjuvanted with CpG was not statistically different than the weight of protected mice pre-exposed to HKx31, whereas mice immunized with PBS, WIV or peptide with IFA and CpG did show a significant (*p* < 0.0001) difference with protected mice. HKx31-specific IgG and HI determination showed that P-V induced minimal amounts of HKx31-specific antibodies, which did not possess any neutralizing capacity (Supplementary Figure S2). Thus, this indicates that the increased recovery was not mediated by cross-reactive neutralizing antibodies, but rather by the increase of CD8^+^ T-cells (and/or CD4^+^ T-cells). Vaccination with P-V mixed with CpG may therefore contribute to the recovery from influenza after heterologous influenza infection.

### Influence of Association Between Peptide and Virosomes on Peptide Association with Dendritic Cells and CD8^+^ T-cell Responses

**Fig. 5:**
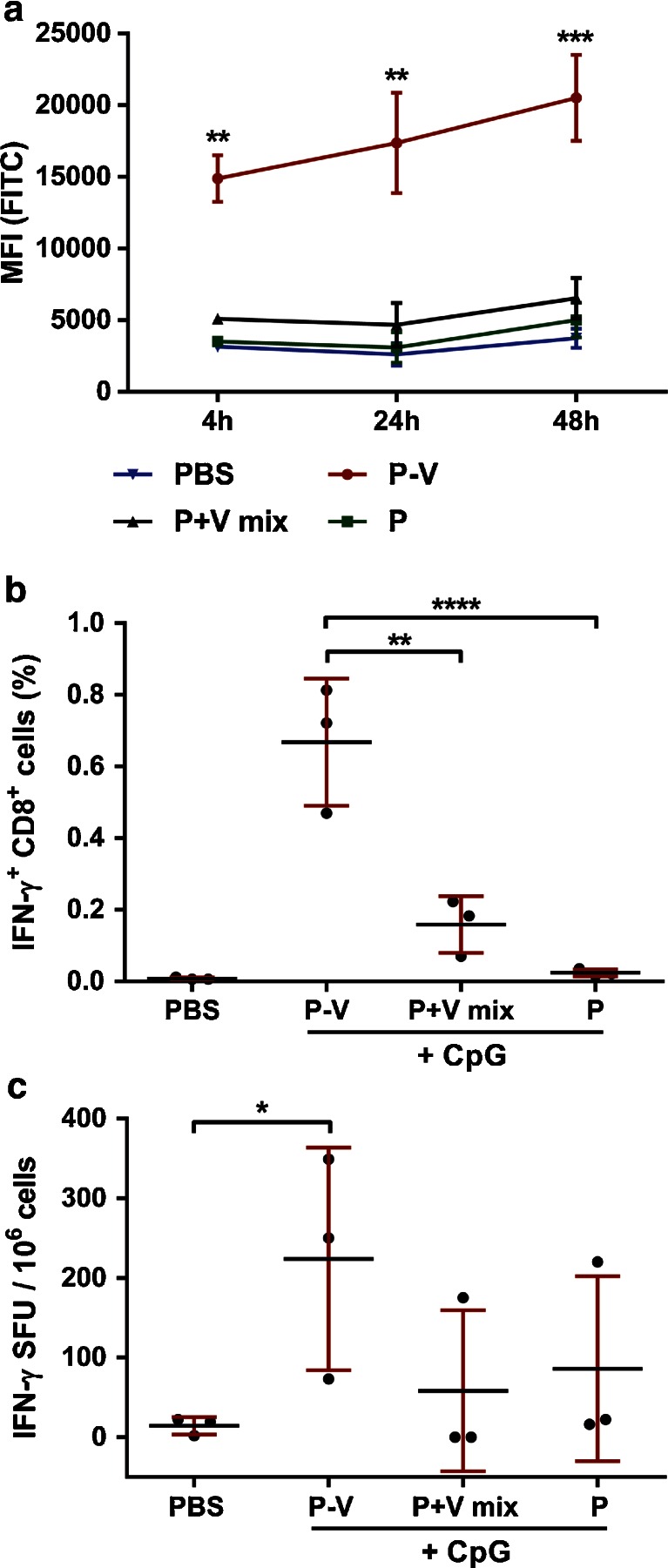
Influence of peptide-virosome association on *in vitro* dendritic cell uptake and *in vivo* T-cell responses. M1_58–66_-FITC peptide uptake by human immature DCs was determined by flow cytometry (**a**). Either PBS, peptide-loaded virosomes (P-V), peptide mixed with empty virosomes (P+V mix) or free peptide (P) were incubated at 37°C with immature DCs for 4, 24 and 48 h. Data represents mean ± SD (*n* = 3) performed with DCs obtained from three different donors. HLA-A2.1 transgenic C57BL/6 mice were immunized twice with aforementioned formulations adjuvanted with CpG. Peptide-specific CD8^+^ T-cell responses were determined in *ex vivo* stimulated splenocytes by flow cytometry (**b**) and ELISPOT (**c**). Data represents mean ± SD (*n* = 3) and is representative of three individual experiments. *p < 0.05, **p < 0.01, ***p < 0.001, ****p < 0.0001.

In order to gain some mechanistic insight into the mode of action of our P-V vaccine, we investigated the importance of peptide association with the virosomes for the induction of peptide-specific CTLs. The delivery concept of virosomes and the importance of peptide association was first assessed *in vitro*. The uptake of peptide antigens by immature DCs was determined *in vitro* for P-V and free peptide mixed with empty virosomes (P+V, Fig. 5a). After 4 h of incubation, P-V showed a significantly (*p* < 0.01) higher peptide association with DCs compared to P+V or free peptide without any carrier. This trend was also observed after 24 and 48 h of incubation, resulting in even larger (*p* < 0.001) differences between P-V and the other formulations. Incubation of P-V with DCs at a temperature of 4°C also showed a significant increase of peptide signal (data not shown), indicating that P-V readily associated with the cell membrane of DCs prior to internalization. This indicates that the virosomes attached themselves to the DC surface most probably by sialic acid binding. Thus, association of the peptide with the virosomes positively affects the antigen association with DCs, proving that virosomes act as a delivery system for the peptide antigen.

In addition to the *in vitro* DC studies, *in vivo* studies in HLA-A2.1 transgenic C57BL/6 mice revealed that association of the peptide with the virosomes is crucial for the induction of peptide-specific CTLs. CD8^+^ T-cell responses were determined in *in vitro* stimulated splenocytes two weeks after the boost vaccination (Fig. 5b). P-V + CpG induced significantly (*p* < 0.0001) higher CTL numbers in mice than the peptide + CpG mixture. When CpG adjuvanted peptide was mixed with empty virosomes, the number of peptide-specific CTLs in the spleen was significantly (*p* < 0.01) lower compared to P-V + CpG. The frequency of IFN-γ producing cells showed a similar trend; only P-V + CpG showed increased IFN-γ spot-forming units compared to peptide mixed with empty virosomes or free peptide alone (Fig. 5c). This suggests that addition of the adjuvant alone to the M_58–66_ peptide is not sufficient to induce peptide-specific T-cell responses. Furthermore, association of the peptide with virosome was not critical for the humoral responses or the T_H1_/T_H2_ balance (Supplemental Figure S3), suggesting that these particular responses are only influenced by the virosome and the presence of an adjuvant.

### Influence of Virosomal Cell Binding and Membrane Fusion Capabilities on the Immunogenicity of Peptide-Loaded Virosomes

**Fig. 6:**
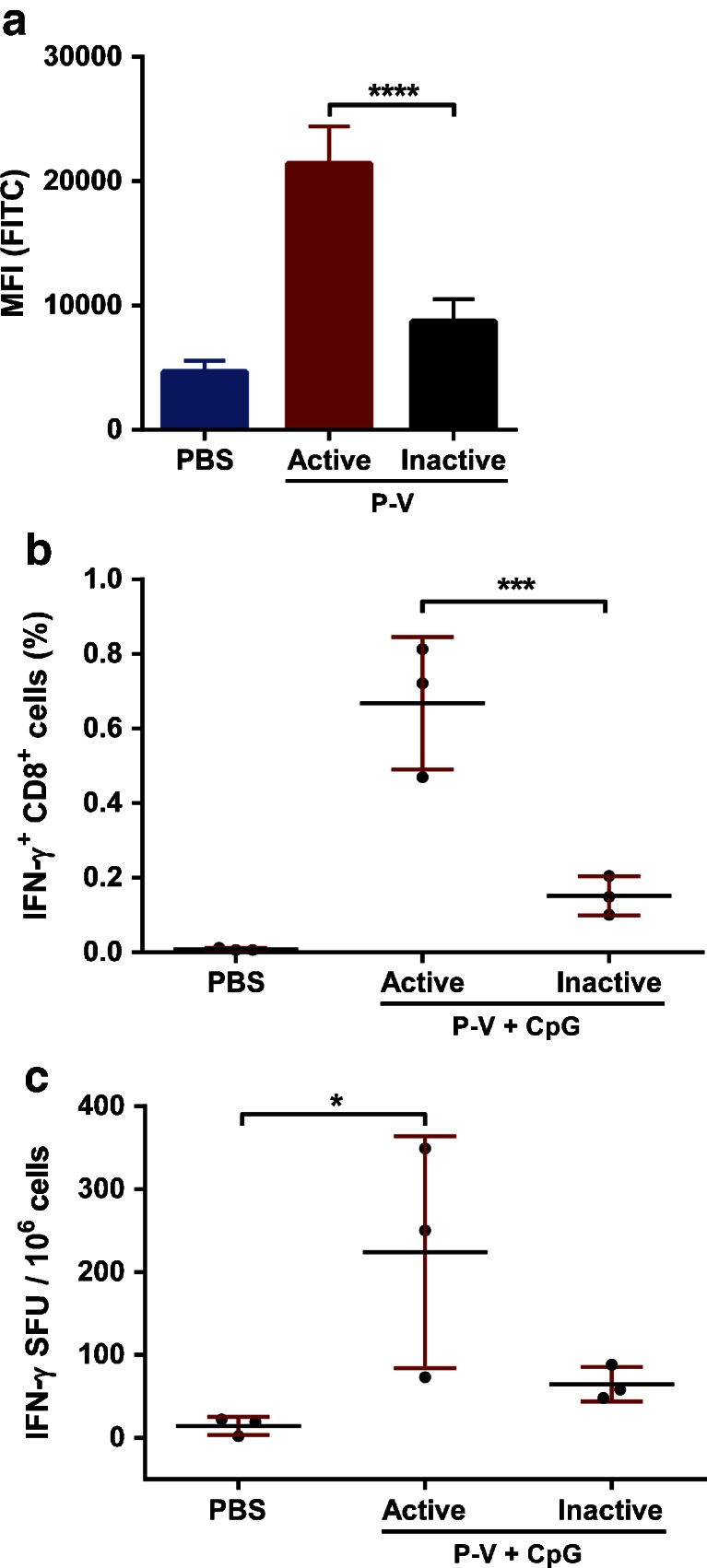
Impact of loss of fusogenic activity on immunogenicity of peptide-loaded virosomes. M1_58–66_-FITC peptide uptake by human immature DCs was quantified for both fusion-active and inactive P-V after 16 h of incubation at 37°C (**a**). Data represents mean ± SD (*n* = 3) performed with DCs obtained from three different donors. HLA-A2.1 transgenic C57BL/6 mice were immunized twice with peptide-loaded virosomes with CpG (Active P-V + CpG) or fusion-inactivated peptide-loaded virosomes with CpG (Inactive P-V + CpG). Peptide-specific CD8^+^ T-cells were subsequently quantified in *ex vivo* stimulated splenocytes by flow cytometry (**b**) and ELISPOT (**c**). Data represents mean ± SD (*n* = 3) and is representative of three individual experiments. *p < 0.05, ***p < 0.001, ****p < 0.0001.

In addition to the role of peptide association, the role of virosomal cell binding and membrane fusion activity in the immunogenicity of peptide-loaded virosomes was studied. Content of virosomes is released into the cytosol of APCs through pH-mediated fusion with the endosomal membrane. First, peptide association by DCs was quantified by flow cytometry after 16 h of incubation with (inactivated) vaccine formulations (Fig. 6a). When P-V were fusion-inactivated, the association of peptide decreased significantly (*p* < 0.0001) compared to fusion-active P-V. This indicates that the inherent cell binding and membrane fusion capabilities of virosomes are needed to ensure efficient uptake by APCs.

Next, we investigated whether the limited uptake of peptide by DCs had an impact on T-cell induction after administering inactivated P-V to mice. HLA-A2.1 transgenic C57BL/6 mice immunized with fusion-inactivated P-V generated significantly (*p* < 0.001) less peptide-specific CD8^+^ T-cells than mice immunized with fusogenic P-V (Fig. 6b). The frequency of IFN-γ producing cells also showed a decreasing trend after inactivation (Fig. 6c), showing that inactivation of the influenza virosomes has a significant negative impact on the immunogenicity of M1_58–66_ peptide associated with the virosome.

To assess the impact of fusion inactivation on the ability of P-V to induce humoral responses, influenza-specific antibody titers were determined in serum. The fusion-inactivated formulation induced a significantly (*p* < 0.0001) lower influenza-specific neutralizing antibody response than the fusion-active P-V (Supplemental Figure S4A). However, the total serum IgG titers were only slightly but significantly (*p* < 0.01) lower for the group receiving fusion-inactivated P-V (Supplemental Figure S4B). This indicates that HA-specific antibodies were unable to inhibit hemagglutination, which could be the effect of reduced antibody avidity or the generation of antibodies recognizing different epitopes. Furthermore, fusion-inactivation of P-V did not affect IgG1 titers, but did negatively affect IgG2c titers (Supplemental Figure S3C and S3D), which indicates a shift to a T_H2_ response.

## DISCUSSION

Current research on universal influenza vaccines is directed at targeting conserved parts of the influenza virion. Aside from B-cell epitopes that can induce broadly-protective neutralizing antibodies, a T-cell component is considered to be an important component of future influenza vaccines (17). Influenza-specific CTLs can increase viral clearance and limit morbidity across multiple influenza strains; moreover, recent studies indicate that cellular responses might be a correlate of protection against pandemic influenza strains (5,18). Inducing potent immune responses against influenza-specific T-cell epitopes, however, is challenging due to the nature of the antigen. Subunit (peptide) vaccines generally possess poor immunogenicity due to the lack of any particulate structure or presence of PAMPs. Virosomes have proven to be an efficient delivery system for peptides in previous studies (10,11,19,20), but generally have low peptide association or encapsulation rates. This makes proper dosage of the peptide antigen difficult; if encapsulation rates are low, a disproportionate amount of virosome material is present in the vaccine. Several alternative production methods have been developed that enhanced peptide encapsulation efficiencies. These included chimeric virosomes (11), virosome lyophilization and subsequent reconstitution (19), or covalent attachment of the peptide (20). However, these methods complicate the production process, and might not be suitable for every virosomal peptide formulation. Thus, the addition of an adjuvant to virosomal peptide formulations could be a viable alternative to raise the immunogenicity of the peptide, without increasing peptide and virosome dose or altering the formulation process.

The selected M1_58–66_ peptide epitope is restricted to the human HLA-A2 serotype. To produce a vaccine that is effective in a global population, several peptide epitopes covering all the HLA serotypes must be included to ensure acceptable coverage. Since few (animal) models currently exist to screen for the various HLA types, we selected a HLA-A2 epitope, which is a common serotype in the Caucasian population and can be tested in HLA-A2.1 transgenic C57BL/6 mice. Thus, in contrast to other preclinical peptide-based vaccine concepts, this concept influenza vaccine could be used directly in humans without changing the peptide antigen.

We demonstrated that the addition of CpG as an adjuvant significantly increased cellular responses in mice immunized with peptide-loaded virosomes. Additionally, CpG skewed antibody responses induced by peptide-loaded virosomes towards the IgG2c isotype. The production of IgG2c antibodies is stimulated during T_H1_ type responses (21), which support the induction of CD8^+^ T-cells (16), which in turn is associated with influenza virus clearance (22). Since our current peptide-loaded virosome production process was inefficient, we opted to mix CpG with our formulation, rather than incorporating it in the virosome, which was previously performed with an avian virosome vaccine (12). Incorporating both CpG and peptide antigen in a virosome potentially could increase immunogenicity due to the simultaneous signal delivery of both adjuvant and antigen. This would be a logical next step for future research.

The interaction between the peptide antigen and the virosome particles was shown to be an important factor for the overall efficacy of the peptide-loaded virosome vaccine, which confirmed an earlier report (10). Additionally, antigen uptake studies with DCs revealed that association of the antigen with the carrier is important for antigen uptake by APCs. While SEC analysis showed that the peptide was indeed associated with the virosomes, the exact localization of the peptide, e.g. in the aqueous inner compartment or the lipid membrane, remains unknown. The localization of peptide antigens in virosomes could have an impact on presentation on MHC-I molecules by APCs (23), which in turn can affect the quality of the immune response, and is therefore a relevant topic for future studies.

Hemagglutinin conformational integrity and activity, mediating virosomal cell binding and membrane fusion, were shown to be crucial for the induction of CD8^+^ T-cell responses. In addition to an earlier report which indicated that fusion activity might affect CTL responses induced by NP_147–155_ peptide-loaded virosomes (10), we demonstrated that virosome fusion inactivation had a profound impact on the capacity of virosomes to deliver peptide to DCs, and on the induction of peptide-specific T-cell responses by peptide-loaded virosomes. The role of fusion activity is not only important for binding and cell entry of virosomes, but also for CTL induction by WIV vaccines (24,25). Furthermore, fusion-inactivation impaired the induction of influenza-specific IgG2c antibodies, which could affect helper T-cell responses. In addition, fusion-inactivation impaired the neutralizing ability of influenza-specific antibodies generated after vaccination with peptide-loaded virosomes significantly, while total influenza-specific IgG levels only were reduced slightly.

To assess the efficacy of the vaccine, HLA-A2.1 transgenic C57BL/6 mice were immunized with CpG-adjuvanted P-V and subsequently challenged with a sublethal heterologous HKx31 influenza infection. The mouse weight loss data shown in this study indicate that the elevated numbers of influenza-specific CTLs after vaccination contributed to the recovery of the mice after heterologous influenza infection, independent of neutralizing antibodies. An increase of circulating CD8^+^ T-cells might however not be enough to provide complete protection against influenza infections. A boost in CD8^+^ T-cells may help clear the virus and improve the rate of recovery of the mice after infection, but is arguably insufficient to prevent the early onset and spread of infection. Slütter *et al.* recently showed the importance of local memory CD8^+^ T-cells in the upper respiratory tract to combat influenza A infections in the early stages (26). This insight implicates that the local induction of respiratory CD8^+^ T-cells could be an important goal for further T-cell based influenza vaccine development. The increased systemic T-cell levels reported in clinical studies might be an indication that elevated local influenza-specific CD8^+^ T-cells in the lungs can provide accelerated recovery and decreased morbidity in influenza-infected patients (5,27).

Aside from CD8^+^ T-cell responses, it has been established that T-cell help (in the form of CD4^+^ T-cells) and B-cell responses should not be overlooked (28), and therefore a vaccine concept that utilizes both T-cell and B-cell responses should be pursued to obtain a universal influenza vaccine (29). Influenza virosomes could be an excellent candidate platform for a cross-protective influenza vaccine, as it is an effective peptide delivery system and a natural carrier of CD4^+^ T-cell and B-cell epitopes.

In conclusion, we demonstrated that peptide-loaded virosomes are able to induce peptide-specific CD8^+^ T-cells. The addition of CpG as an adjuvant further increased the efficacy of peptide-loaded virosomes. Aside from a greater number of peptide-specific CD8^+^ T-cells, CpG adjuvanted P-V also induced T-cell help and influenza-specific antibodies. Peptide-virosome association and virosome fusion activity are important factors for the effectiveness of P-V. The synergistic effect of virosome particles, fusion activity and CpG make a potent combination to increase the immunogenicity of peptide antigens. Thus, peptide-loaded virosomes are a promising approach for the development of a cross-protective influenza vaccine.

## Electronic supplementary material

Below is the link to the electronic supplementary material.

Supplementary Figure S1 Contribution of WIV to peptide-specific T-cell responses and total influenza-specific IgG titers after immunization. HLA-A2.1 transgenic C57BL/6 mice were immunized twice with 1 μg of M1_58–66_ peptide formulated in either virosomes (P-V) or with whole inactivated influenza virus (WIV) with CpG (P + WIV). Mice were immunized with PBS as negative control. Two weeks after the final vaccination, peptide-specific CD8+ T-cell responses in *ex vivo* stimulated splenocytes were determined using flow cytometry (A) and ELISPOT (B). Influenza-specific total IgG titers in sera from mice (C). Data represents mean ± SD (n = 6). *p < 0.05, **p < 0.01; n.d., not detectable. (GIF 32 kb)

High Resolution Image (TIFF 797 kb)

Supplementary Figure S2 Minimal HKx31-specific humoral response induced by peptide-loaded virosomes. Influenza HKx31-specific total IgG titers (A) and HI titers (B) in sera from mice after immunization with either PBS, HKx31 virus or P-V. Data represents mean ± SD (n = 6). n.d., not detectable. (GIF 12 kb)

High Resolution Image (TIFF 336 kb)

Supplementary Figure S3 Effect of association between peptide and virosomes on humoral responses. Mice sera were analyzed for HI (A) and total IgG (B) titers. IgG1 (C) and IgG2c (D) isotypes were also determined from sera. Data represents mean ± SD (n = 3) and is representative of three individual experiments. n.d., not detectable. (GIF 34 kb)

High Resolution Image (TIFF 1354 kb)

Supplementary Figure S4 Effect of fusogenic activity of virosomes on influenza-specific antibodies. Influenza-specific HI titers (A) and total IgG titers (B), and antibody isotypes IgG1 (C) and IgG2c (D) titers in sera from mice. Data represents mean ± SD (n = 3) and is representative of three individual experiments. **p < 0.01, ****p < 0.0001. (GIF 36 kb)

High Resolution Image (TIFF 1333 kb)

## ABBREVIATIONS

CpG: CpG-ODN 1826
IFA: Incomplete Freund’s adjuvant
P-V: Peptide-loaded virosomes
