# Supplementary figures and images for: Influenza T-cell Epitope-Loaded Virosomes Adjuvanted with CpG as a Potential Influenza Vaccine
Source: Pharm Res. 2014 Oct 25;32(4):1505–15. doi: 10.1007/s11095-014-1556-3 (PMC4356889; doi:10.1007/s11095-014-1556-3)

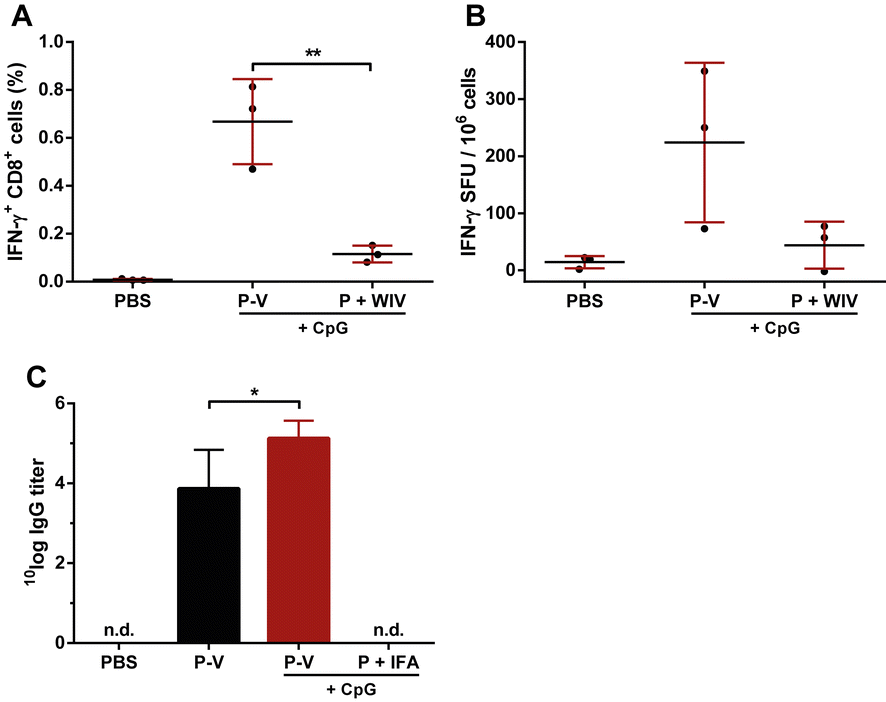

Supplement: Supplementary file 1 — Contribution of WIV to peptide-specific T-cell responses and total influenza-specific IgG titers after immunization. HLA-A2.1 transgenic C57BL/6 mice were immunized twice with 1 μg of M158–66 peptide formulated in either virosomes (P-V) or with whole inactivated influenza virus (WIV) with CpG (P + WIV). Mice were immunized with PBS as negative control. Two weeks after the final vaccination, peptide-specific CD8+ T-cell responses in ex vivo stimulated splenocytes were determined using flow cytometry (A) and ELISPOT (B). Influenza-specific total IgG titers in sera from mice (C). Data represents mean ± SD (n = 6). *p < 0.05, **p < 0.01; n.d., not detectable. (GIF 32 kb) [file 11095_2014_1556_Fig7_ESM.gif]

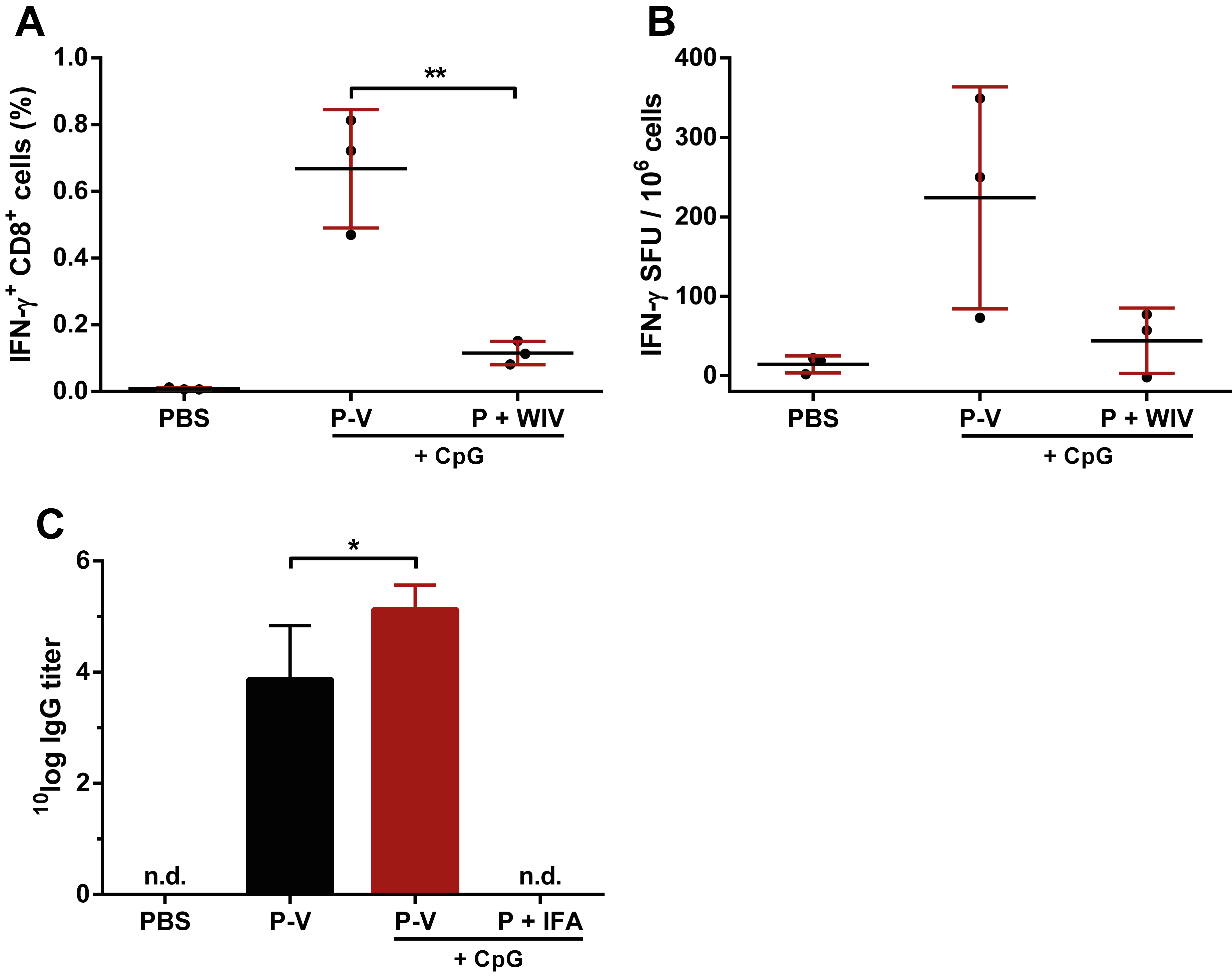

Supplement: Supplementary file 2 — High Resolution Image (TIFF 797 kb) [file 11095_2014_1556_MOESM1_ESM.tif]

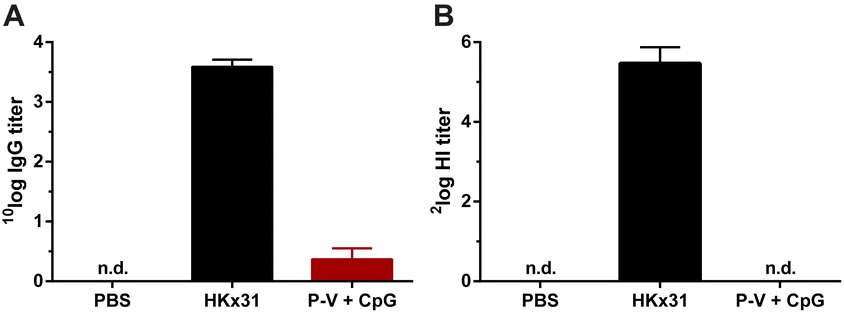

Supplement: Supplementary file 3 — Minimal HKx31-specific humoral response induced by peptide-loaded virosomes. Influenza HKx31-specific total IgG titers (A) and HI titers (B) in sera from mice after immunization with either PBS, HKx31 virus or P-V. Data represents mean ± SD (n = 6). n.d., not detectable. (GIF 12 kb) [file 11095_2014_1556_Fig8_ESM.gif]

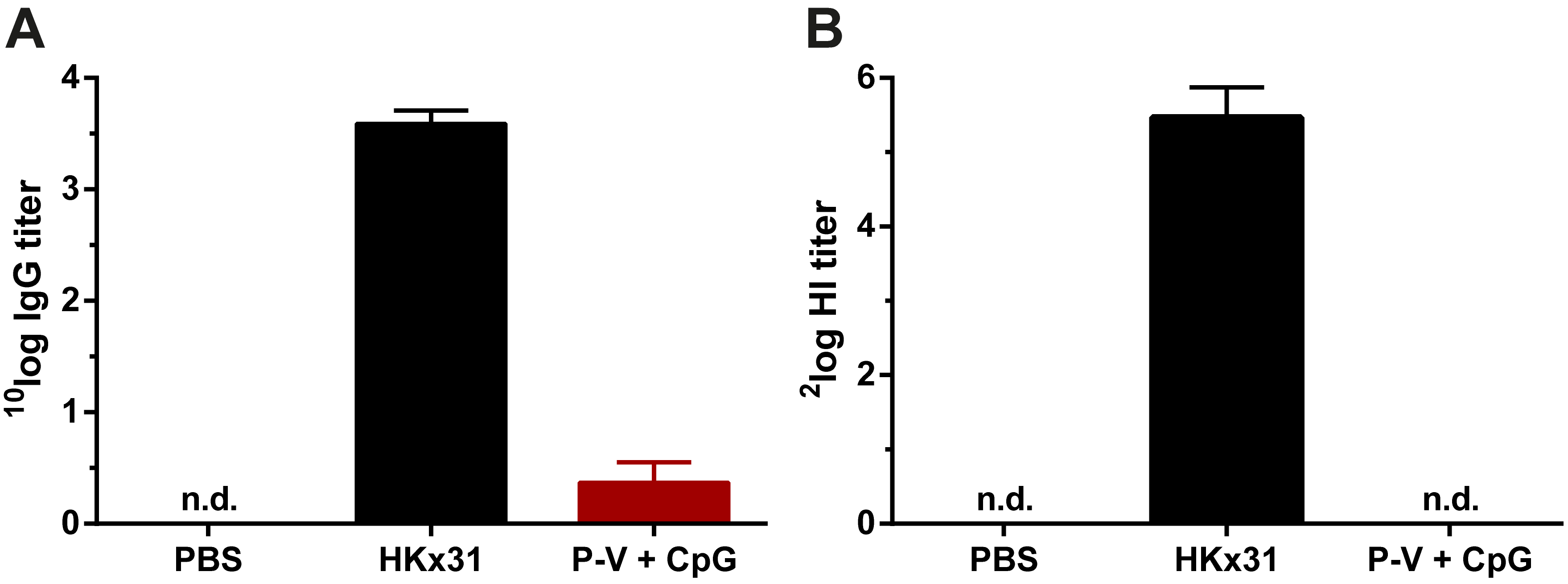

Supplement: Supplementary file 4 — High Resolution Image (TIFF 336 kb) [file 11095_2014_1556_MOESM2_ESM.tif]

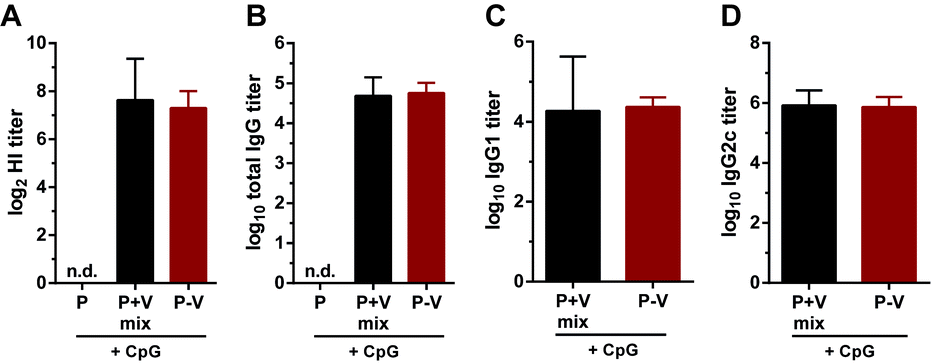

Supplement: Supplementary file 5 — Effect of association between peptide and virosomes on humoral responses. Mice sera were analyzed for HI (A) and total IgG (B) titers. IgG1 (C) and IgG2c (D) isotypes were also determined from sera. Data represents mean ± SD (n = 3) and is representative of three individual experiments. n.d., not detectable. (GIF 34 kb) [file 11095_2014_1556_Fig9_ESM.gif]

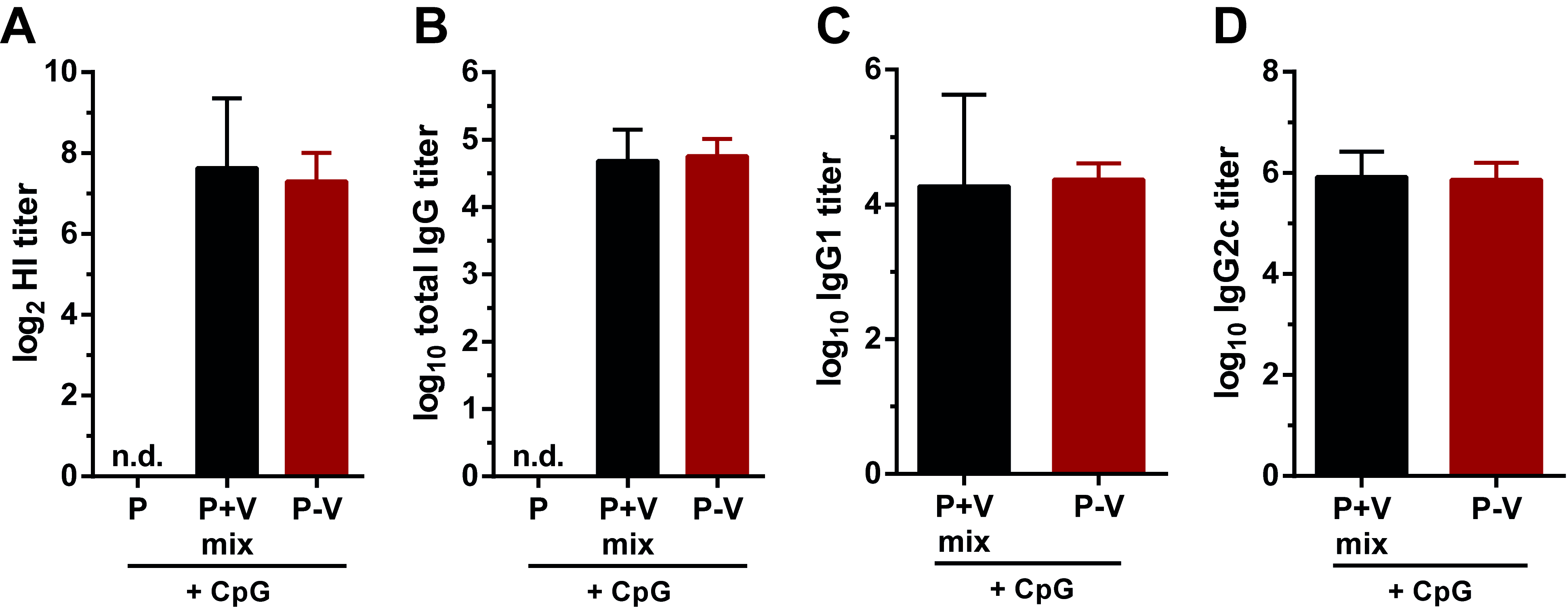

Supplement: Supplementary file 6 — High Resolution Image (TIFF 1354 kb) [file 11095_2014_1556_MOESM3_ESM.tif]

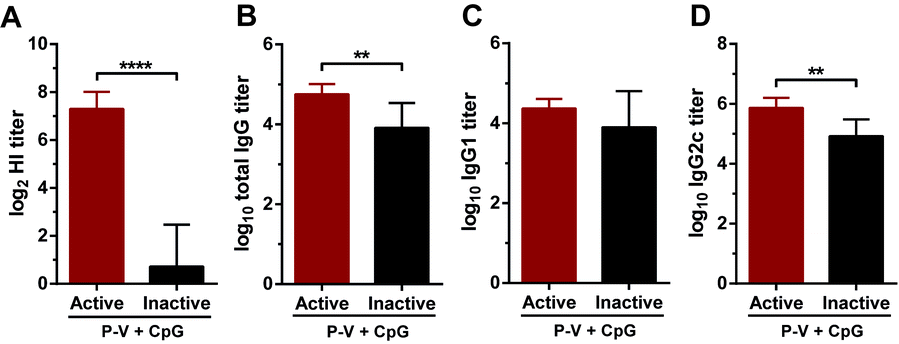

Supplement: Supplementary file 7 — Effect of fusogenic activity of virosomes on influenza-specific antibodies. Influenza-specific HI titers (A) and total IgG titers (B), and antibody isotypes IgG1 (C) and IgG2c (D) titers in sera from mice. Data represents mean ± SD (n = 3) and is representative of three individual experiments. **p < 0.01, ****p < 0.0001. (GIF 36 kb) [file 11095_2014_1556_Fig10_ESM.gif]

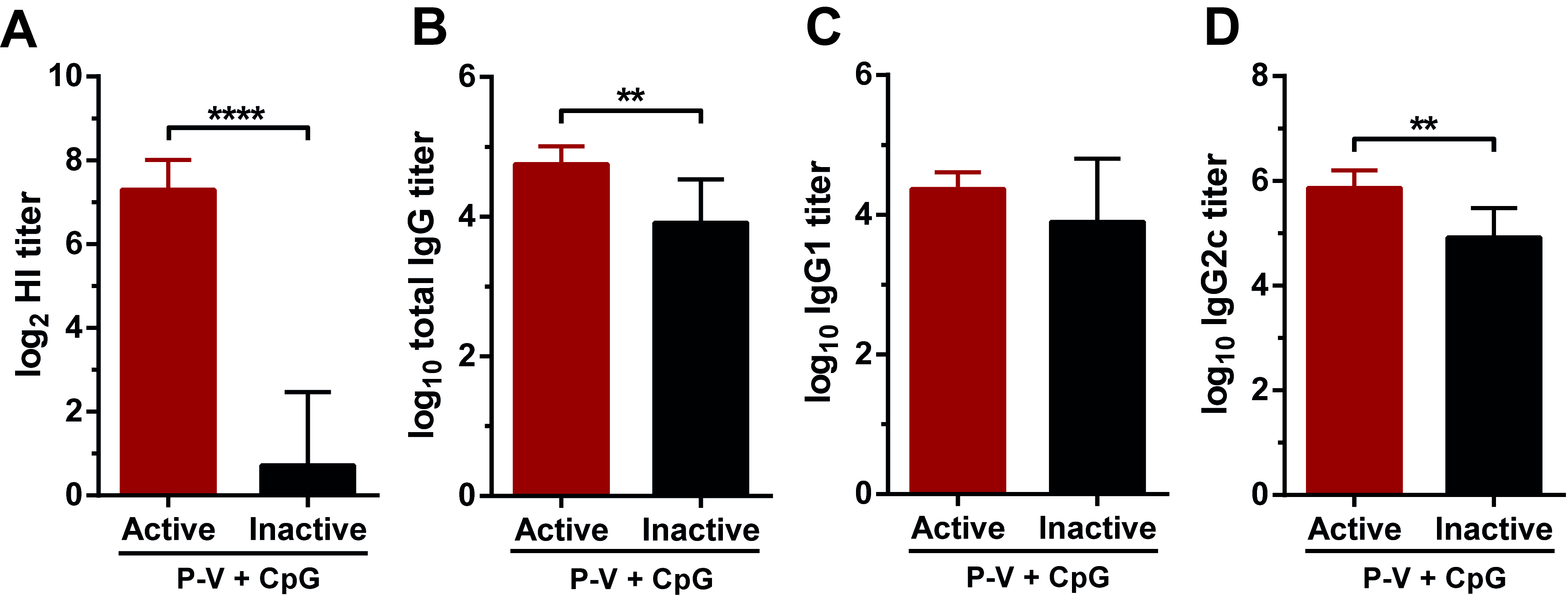

Supplement: Supplementary file 8 — High Resolution Image (TIFF 1333 kb) [file 11095_2014_1556_MOESM4_ESM.tif]
